# Disruption of the microbiota across multiple body sites in critically ill children

**DOI:** 10.1186/s40168-016-0211-0

**Published:** 2016-12-29

**Authors:** Matthew B. Rogers, Brian Firek, Min Shi, Andrew Yeh, Rachel Brower-Sinning, Victoria Aveson, Brittany L. Kohl, Anthony Fabio, Joseph A. Carcillo, Michael J. Morowitz

**Affiliations:** 1 Department of Surgery, School of Medicine, University of Pittsburgh, Pittsburgh, USA; 2 Division of Pediatric General and Thoracic Surgery, Children’s Hospital of Pittsburgh of UPMC, Pittsburgh, USA; 3 Division of Pediatric Critical Care Medicine, Children’s Hospital of Pittsburgh of UPMC, Pittsburgh, USA; 4 Department of Epidemiology, Graduate School of Public Health, University of Pittsburgh, Pittsburgh, USA; 5 Department of Critical Care Medicine, School of Medicine, University of Pittsburgh, Pittsburgh, USA; 6 Division of Pediatric Surgery, Children’s Hospital of Pittsburgh of UPMC, 4401 Penn Avenue, 7th Floor Faculty Pavilion, Pittsburgh, PA 15244 USA

**Keywords:** Microbiome, Microbiota, Critical illness, Pediatric critical care, Microbial diversity, Dominance, Site specificity, Antibiotics, Nosocomial infection, Sepsis

## Abstract

**Background:**

Despite intense interest in the links between the microbiome and human health, little has been written about dysbiosis among ICU patients. We characterized microbial diversity in samples from 37 children in a pediatric ICU (PICU). Standard measures of alpha and beta diversity were calculated, and results were compared with data from adult and pediatric reference datasets.

**Results:**

Bacterial 16S rRNA gene sequences were analyzed from 71 total tongue swabs, 50 skin swabs, and 77 stool samples or rectal swabs. The mean age of the PICU patients was 2.9 years (range 1–9 years), and many were chronically ill children that had previously been hospitalized in the PICU. Relative to healthy adults and children, alpha diversity was decreased in PICU GI and tongue but not skin samples. Measures of beta diversity indicated differences in community membership at each body site between PICU, adult, and pediatric groups. Taxonomic alterations in the PICU included enrichment of gut pathogens such as *Enterococcus* and *Staphylococcus* at multiple body sites and depletion of commensals such as *Faecalibacterium* and *Ruminococcus* from GI samples. Alpha and beta diversity were unstable over time in patients followed longitudinally. We observed the frequent presence of “dominant” pathogens in PICU samples at relative abundance >50%. PICU samples were characterized by loss of site specificity, with individual taxa commonly present simultaneously at three sample sites on a single individual. Some pathogens identified by culture of tracheal aspirates were commonly observed in skin samples from the same patient.

**Conclusions:**

We conclude that the microbiota in critically ill children differs sharply from the microbiota of healthy children and adults. Acknowledgement of dysbiosis associated with critical illness could provide opportunities to modulate the microbiota with precision and thereby improve patient outcomes.

**Electronic supplementary material:**

The online version of this article (doi:10.1186/s40168-016-0211-0) contains supplementary material, which is available to authorized users.

## Background

Studies of the human microbiome have generally targeted healthy volunteers or individuals with chronic diseases. With the exception of newborn infants, relatively few studies have characterized the microbiota of hospitalized patients [1–8], particularly those that are critically ill. This is an important knowledge gap since dysbiosis may contribute to adverse outcomes among ICU patients and the cost of ICU care [9]. Obvious potential causes for disruption of the ICU microbiome include antibiotics [10], dietary changes [11], and exposure to nosocomial pathogens [12].

What is known about colonization patterns in ICU patients comes largely from older, culture-dependent studies. It has been demonstrated that risk of colonization by pathogens increases over time in the ICU [13–17] and that colonization increases the probability of developing a subsequent infection [5, 18–22]. Generally, these older studies were designed to confirm the presence or absence of specific pathogens (e.g., *Staphylococcus aureus*). As a result, we lack a global understanding of how the microbiota changes during critical illness. Recently, we and others have demonstrated in critically ill adult patients that an ICU stay is associated not only with appearance of pathogens but also loss of commensal organisms from multiple body sites [2, 23]. However, little has been published on this topic with regard to critically ill children.

Here, we characterized skin, oral, and fecal microbial communities in a cohort of critically ill children. We selected these body sites because bacterial populations at these locations may impact the risk of hospital-acquired infections, e.g., pneumonia- and catheter-related sepsis [24–26]. In most cases, the observed temporal and spatial dynamics of microbial colonization differed significantly from patterns observed in healthy children and adults, likely reflecting both underlying illness and the corresponding treatments (e.g., antibiotic administration). Data from this pediatric cohort appear to validate recent reports [2] that ICU-associated dysbiosis is generally characterized by loss of diversity, loss of body site-specificity, and presence of pathogens at high levels of abundance.

## Results

### Microbial diversity among critically ill children differs significantly from healthy adults and children

We analyzed 16S rRNA gene pyrosequencing data from 71 tongue swabs, 50 skin swabs, and 77 GI samples (37 fecal samples, 40 rectal swabs) collected from 37 patients over 39 pediatric ICU (PICU) admissions [27]. Samples with below 200 reads following clustering were removed from downstream analysis. A single set of samples was analyzed for 23 subjects, whereas longitudinal series of samples were analyzed for the remaining 14 subjects (detailed metadata provided in Additional files 1 and 2). The average number of sequencing reads per sample was 2980 (463–41,180 reads). The mean age of the patients was 2.9 years (range 1–9). Of these patients, 86.5% were chronically ill and 45.9% had a PICU stay within the prior 6 months (mean number of days 19.8 days, range 2–64 days). Nearly all subjects received antibiotics (89.2%) and required mechanical ventilation (94.6%).

We compared our dataset to published 16S rRNA gene sequences obtained from the tongue dorsum, stool, and skin (antecubital fossa) of 227 healthy adult volunteers in the Human Microbiome Project [28]. Because age impacts the microbiota [29], we also sought comparisons of the PICU data with healthy age-matched children. To our knowledge, however, there are no analogous public datasets containing microbial sequencing data obtained simultaneously from multiple body sites from individual children. Therefore, we also analyzed GI, tongue, and skin samples collected from 13 children ages 1–9 (Additional file 2) admitted to our hospital for brief observation after minor trauma (mean age 4.6 years; average number of pyrosequencing reads per sample 2175; range 451–4784). These patients had not received antibiotics in the prior 6 months, and they received a regular diet during their hospitalization and thus were considered a valid reference group for comparison with PICU patients.

**Fig. 1:**
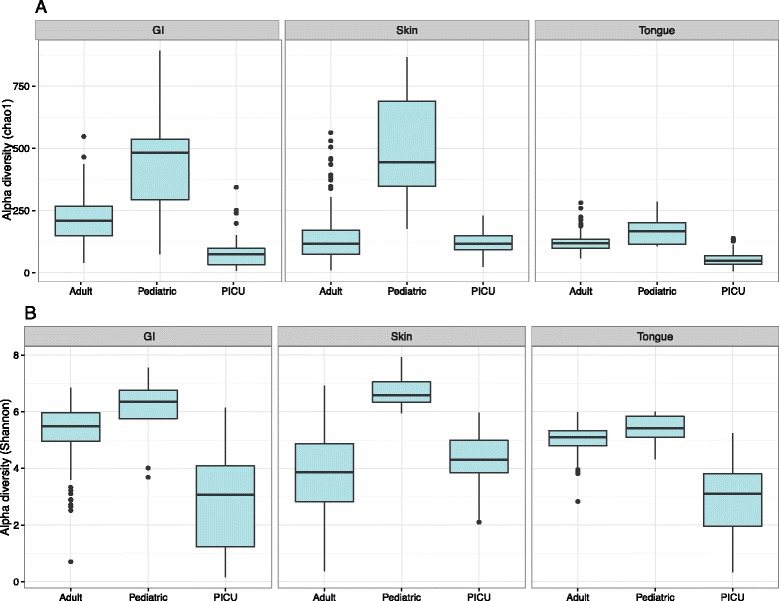
Alpha diversity comparisons of microbial communities of PICU patients, healthy adults, and healthy children. Shown are **a** calculated Chao1 species richness indices for PICU, pediatric, and reference samples and **b** the calculated Shannon species evenness predictions for the same groups at each site

Alpha diversity is an ecological measure of how many taxonomic groups are present within each sample and whether the abundance of these groups is evenly distributed [30]. Compared to adult and pediatric samples, PICU GI and oral samples were significantly reduced in alpha diversity, using either the Chao1 diversity metric or the Shannon entropy metric (non-parametric test, all *p* values <0.003) (Fig. 1). PICU skin samples were significantly lower in diversity compared to pediatric samples, but not HMP samples (non-parametric test, all *p* values <0.003).

**Fig. 2:**
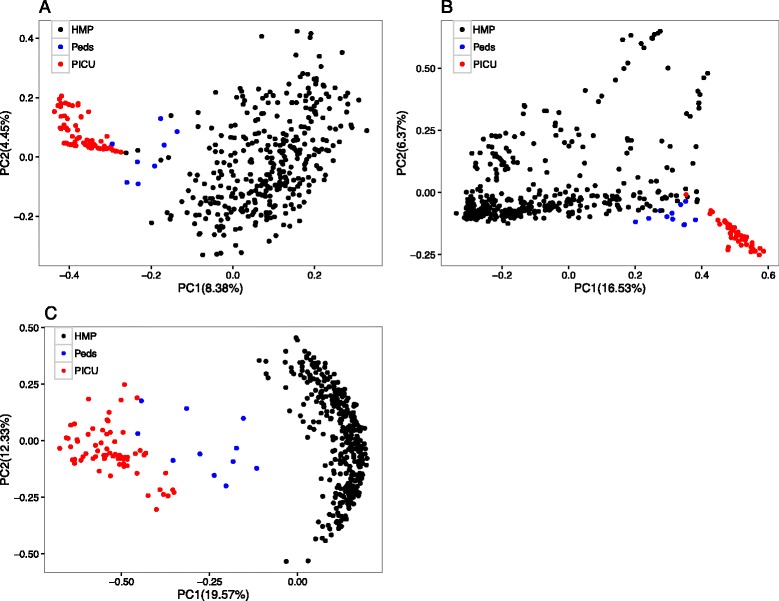
Beta diversity comparisons of microbial communities of PICU patients, healthy adults, and healthy children. Displayed are principal coordinate analyses (**a** gut; **b** skin; **c** tongue) of abundance Jaccard distances between samples from PICU patients, healthy children, and healthy adults. *Axis labels* indicate the proportion of variance explained by each principal coordinate axis

To characterize similarities and differences in the composition of microbial communities, we calculated two measures of beta diversity, weighted UniFrac distances and Jaccard indices (Fig. 2; see also supporting figures in Additional file 3). Community composition at each body location was significantly different between PICU populations and both reference sets (Jaccard distances, PERMANOVA *p* < 0.001).

To further define the PICU-associated microbiota, LEfSe [31] was used to identify taxa at a given body site that were either enriched or depleted specifically in PICU samples (Additional file 4) (LEfSe *p* values <0.05 for all taxa listed as enriched or depleted). At the phylum level, PICU oral samples were enriched for Bacteroidetes, GI samples were enriched for Firmicutes, Actinobacteria, and Fusobacteria, and skin samples were enriched for Firmicutes and Bacteroidetes. At the genus level, PICU samples were enriched for two common ICU pathogens (*Enterococcus* and *Staphylococcus*) at all three body sites and enriched for *Pseudomonas* in GI and oral samples. Additional features of the PICU microbiome are displayed in Additional files 4 and 5, including the sharp enrichment of *Capnocytophaga* in oral and skin samples. Importantly, PICU samples were found to be depleted of several commensals identified in healthy adults and children. Depleted taxa within PICU GI samples included several anaerobes associated with gut health, e.g., *Ruminococcus*, *Roseburia*, and *Faecalibacterium*. PICU oral samples were depleted of commensals such as *Rothia* and *Haemophilus*, and PICU skin samples were also depleted of *Haemophilus*.

Phylogenetic analysis of OTUs corresponding to *Pseudomonadaceae* indicated that some of the observed OTUs could be assigned to a contaminant in control samples that most closely maps to either *Pseudomonas putida* or *Pseudomonas fluorescens*. However, most of the observed *Pseudomonadaceae* OTUs in patient samples mapped to a clade of *Pseudomonas aeruginosa*, a well-recognized human pathogen that was also isolated by the hospital clinical microbiology laboratory in tracheal aspirates obtained from several of the study subjects (Additional file 2). OTUs from the genus *Capnocytophaga* were most commonly from taxa previously observed in the human oral cavity: *C. leadbetteri*, *C. sputigena*, and *C. gingivalis*.

### ICU samples are characterized by dominant pathogens and loss of site specificity

**Fig. 3:**
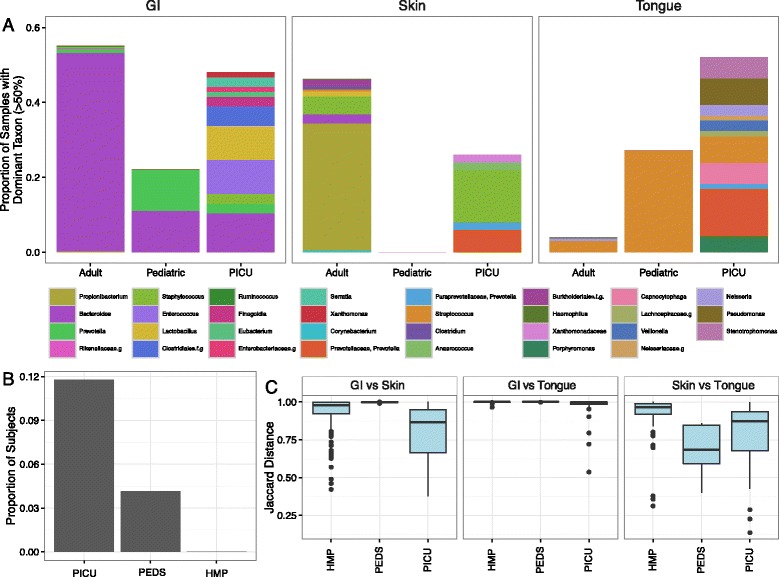
Dominant pathogens and loss of site specificity in PICU samples. **a** Proportion of overall samples in which more than 50% of sequencing reads are derived from a single dominant bacterial taxon. *Colored stacked bars* indicate the identity of the dominant taxa. Many dominant genera in the adult and pediatric groups are known commensals, whereas many dominant taxa identified in PICU samples are pathogenic. **b** Proportion of subjects with *any* dominant genus present on three body sites simultaneously. Many PICU subjects harbored three *distinct* dominant pathogens simultaneously at the three body sites studied. **c** Boxplot of abundance Jaccard distances between samples collected on the same date from PICU, pediatric, and adult subjects. Shown are distances between GI and skin samples from the same individual, GI and tongue samples from the same individual, and skin and tongue samples from the same individual. In all comparisons except skin vs. tongue, we found the median distance between samples to be significantly reduced in PICU patients

The presence of a “dominant” pathogen within fecal samples has been identified as a risk factor for subsequent infections caused by that same organism [5, 21]. To our knowledge, our recent study of critically ill adults was the first to quantify the number of samples in the Human Microbiome Project with dominant taxa [28]. We defined dominance as the presence of a single taxon with relative abundance exceeding 50%. Here, we found that the percentage of GI and tongue samples with dominant taxa was also higher among PICU samples than adult or pediatric samples (Fig. 3a). Although healthy adult samples did contain many dominant taxa, these taxa were typically normal commensals such as *Propionibacterium* (skin) and *Bacteroides* (gut). By contrast, dominant taxa in the PICU were more likely to be pathogenic, e.g., *Enterococcus* (GI), *Staphylococcus* (skin), *Porphyromonas* (tongue), *Pseudomonas* (tongue), *Capnocytophaga* ﻿(tongue), and *Stenotrophomonas* (tongue). Remarkably, the PICU population included many subjects in whom all examined body sites simultaneously harbored dominant organisms (most commonly with different dominant taxa at each location). This pattern was observed only rarely among healthy children and adults (Fig. 3b).

Another well-described feature of the human microbiome is site specificity. It has been demonstrated repeatedly that microbial community membership typically varies significantly between body sites [32]. In our study of critically ill adults, we noted that pathogenic taxa were commonly present simultaneously at multiple body sites at relatively high abundance. Among PICU patients, we observed a similar phenomenon. The genus most commonly observed simultaneously at all three body sites in both PICU and adult datasets was *Prevotella*, but this phenomenon was also observed in the PICU with *Staphylococcus*, *Stenotrophomonas*, and *Porphyromonas*.

To better define the loss of site specificity in the ICU, we calculated abundance Jaccard distances between microbial communities for each pair of body locations (gut vs. skin, gut vs. tongue, and skin vs. tongue) from all individuals with complete sets of GI, skin, and oral sequencing data (Fig. 3c). Accounting for sequencing failures from one or more body sites, we identified 120 healthy adults, 33 PICU subjects, and five healthy children with at least one complete set of contemporaneous samples collected from three body sites on the same day. We found that the distance between body sites was significantly reduced in PICU patients relative to HMP subjects for all comparisons between sites (Welch’s two-sample *t* test, *p* values <0.05). The distances between GI vs. tongue and GI vs. skin samples from PICU patients were also reduced relative to healthy children, although the distance between tongue vs. skin samples was not reduced significantly. Interestingly, some paired distances of healthy children were reduced relative to HMP subjects, suggesting that age contributes partly to the development of site-specific microbial communities.

### The microbiota often but not always changes radically during PICU admission

**Fig. 4:**
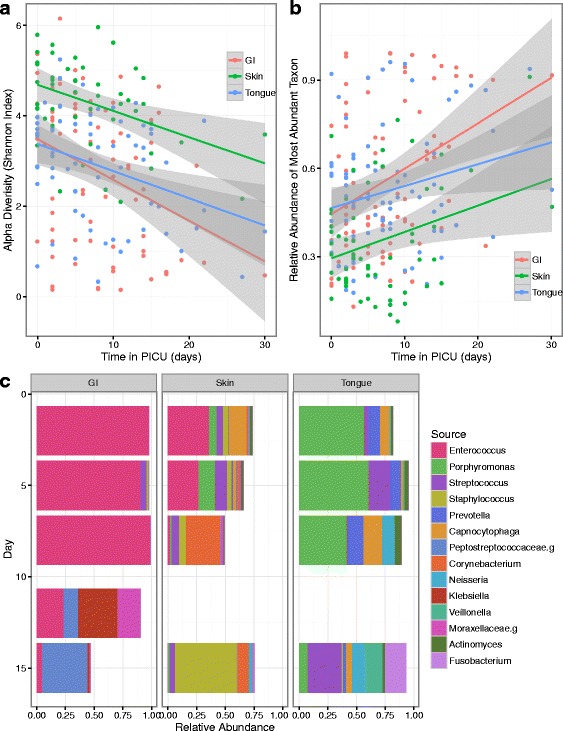
Temporal changes in the site-specific microbiota of PICU patients. **a** Temporal changes in the median Shannon diversity index of study subjects followed longitudinally during their PICU admission. All *p* values <0.05. **b** Temporal changes in relative abundance of most abundant taxa in PICU patients. With time in the ICU, dominant taxa become more prevalent at each body site. All *p* values <0.05. **c** Temporal and spatial variation of the microbiota in a single individual. Shown here are microbial profiles for a 2-year-old chronically ill child with enterococcal sepsis. With time and antibiotics, the dysbiosis seen on admission resolves. The enterococcal populations at all three body sites nearly disappear, and the skin and oral communities also adopt configurations more typical of healthy individuals (e.g., *Staphylococcus* on the skin and *Streptococcus* and *Neisseria* in the mouth)

The mean alpha diversity of samples correlated inversely with days spent in the PICU (Shannon diversity index, GI *r* ^*2*^ = 0.115; skin *r* ^*2*^ = 0.173; oral *r* ^*2*^ = 0.122) (Fig. 4a). A corresponding finding was an increase in the prevalence of dominant taxa over time among the PICU samples (GI *r* ^*2*^ = 0.134; skin *r* ^*2*^ = 0.093; tongue *r* ^*2*^ = 0.056) (Fig. 4b). In some subjects but not all, alpha diversity recovered near the end of the ICU admission (see supplemental figure in Additional file 6).

To examine the taxonomic stability of the ICU microbiome over time, we calculated binary Jaccard distances for all samples collected at adjacent time points from the same individual and same body site, as well as binary Jaccard distances between samples collected on the same study date from unrelated individuals (see the figure in Additional file 7). Overall, samples from different individuals taken on the same date were significantly different from samples collected on adjacent dates from the same individuals (GI; Wilcoxon’s rank sum test; *p* value <2.2e−16; oral *p* value <2.2e−16; skin *p* value = 5.845e−09). Most samples were more similar to other samples from the same individual than samples from other individuals, as also shown in a similar analysis of gut microbiota in infants [33]. However, we observed a much broader range of values for intra-individual comparisons in the PICU than was observed in healthy children. This is evidence that dynamic temporal changes exist in the microbiota of ICU patients, as major differences were observed in a subset of samples collected at adjacent dates from the same patient.

The potential value of analyzing temporal and spatial variation in the microbiota is illustrated by time series analysis of patient 6 (Fig. 4c). This 2-year-old child was admitted to the ICU with sepsis. Blood cultures on admission were positive for growth of *Enterococcus*, *Acinetobacter*, and *Staphylococcus* (latter two organisms suspected to be skin contaminants from venipuncture). Interestingly, microbiota profiling demonstrated *Enterococcus* at two body sites shortly after admission (relative abundance GI 97.9%, skin 35.8%, and tongue 0%), and three body sites 3 days later (GI 90.6%, skin 26.9%, and tongue 0.005%). Early tongue samples were dominated by *Porphyromonas*. Over time, after the initiation of antibiotics and resolution of clinical symptoms, the dominant populations of *Enterococcus* and *Porphyromonas* disappeared and alpha diversity recovered.

### Associations between clinical information and configuration of the microbiota

We sought to characterize associations between positive microbiology culture results and microbiota profiles. Of the 39 PICU admissions, 27 were marked by at least one positive culture (see metadata in Additional file 2). In contrast to the small group of blood, wound, and urine cultures, only the group of 31 positive tracheal aspirates (TAs) was large enough to assess for associations with the microbiota. The pathogen isolated most frequently in TAs was *P. aeruginosa*; *S. aureus*, *Serratia marcescens*, and *Stenotrophomonas maltophilia* were also observed frequently. We compared microbiota profiles of patients with positive TAs to profiles from patients without positive cultures from any body location. Interestingly, we found that patients with TAs positive for *Serratia* (1.5 vs. <1%, Wilcoxon’s rank sum test, *p* value = 0.01959) and *Stenotrophomonas* (3.3 vs. <1%, Wilcoxon’s rank sum test, *p* value = 0.03707) harbored skin swabs (collected within 2 days of the positive culture) with significantly higher abundance of these same organisms than patients without positive cultures; the same relationship did not hold for other genera, and it did not hold true for GI or tongue samples. These correlations between tracheal aspirate and skin colonization patterns may represent direct inoculation of the anterior chest wall via aerosolization. *Pseudomonas* was extremely common on PICU tongue swabs but was present at varying levels of abundance; a relative abundance of *Pseudomonas* greater than 0.5% on a tongue swab (collected within 2 days of the positive culture) was highly associated with the presence of a TA positive for *P. aeruginosa*, although this association did not reach statistical significance (25% of positive samples vs. 0% samples negative for *Pseudomonas*; *χ* ^2^ = 1.8863, df = 1, *p* value = 0.1696).

## Discussion

In this observational analysis of the microbiota in critically ill children, we documented loss of alpha diversity and changes in beta diversity present relative to healthy children and adults. The children in this study demonstrated an abundance of nosocomial pathogens across all body sites and a reduced abundance of gut commensals such as *Faecalibacterium*. Additional features of dysbiosis observed in our study population were loss of site specificity and dominance by pathogenic taxa. These findings likely reflect numerous variables including multiple antibiotic exposures, derangements in host physiology, and/or the presence of nosocomial pathogens. More research will be required to determine the clinical relevance of these findings, e.g., the presence of a pathogen at multiple body sites may represent an actionable risk factor for nosocomial infection.

Prior investigations of the ICU microbiome have generally been restricted to a single body site (commonly the gut) and have suffered from limitations of culture-based approaches. Still, these studies have provided a rationale for therapeutic strategies to restore missing commensals and/or eradicate pathogens in critically ill patients. Two general approaches have been investigated to “manipulate” the ICU microbiome: probiotic administration and selective decontamination of the skin, gut, or oropharynx. Each of these strategies carries documented risks (e.g., antibiotic resistance) and benefits (e.g., reduced incidence of nosocomial infections) [34–36]. Importantly, these strategies have been implemented without personalized knowledge of the microbiota in the patients receiving these interventions. We suggest that such strategies may benefit from an improved understanding of bacterial colonization patterns in the ICU. Even with conventional antibiotic therapy, it could be useful to profile the microbiota in real-time before, during, and after therapy.

## Conclusions

This study adds to an emerging literature describing dysbiosis in the ICU. Multiple groups have now reported on loss of diversity, loss of site specificity, disappearance of commensals, and appearance of pathogens in ICU patients. In parallel, basic and translational studies have clearly demonstrated links between the microbiota and immune function [37], systemic inflammation [38, 39], metabolism [40], CNS function [41, 42], and circadian rhythm [43]. Taken together, these studies should contribute to an increased awareness among clinicians that critical illness is associated with disturbances in the microbiota. It is reasonable to surmise that ICU dysbiosis impacts the risk of short- and long-term adverse events in the ICU including undernutrition, nosocomial infection, and neurocognitive outcomes. Thus, we are hopeful that real-time monitoring and modulation of microbial populations across diverse body sites can improve clinical outcomes in the ICU.

## Methods

### Subject recruitment and participation

Thirty-seven children (ages 1–9) admitted to the Pediatric ICU (PICU) of the Children’s Hospital of Pittsburgh of UPMC (August 2012 to November 2013) were enrolled by convenience sampling according to a protocol approved by the Institutional Review Board of The University of Pittsburgh (#11120410). Parents of all study subjects provided written consent for participation in the study and for publication of study results. All PICU patients in this age range were eligible for inclusion except patients that were not expected to receive enteral feedings during their PICU admission due to intestinal disorders. Parents from 115 total PICU patients were approached for possible participation, but many refused participation. A single set of patient samples was collected from most participating study subjects within 3 days of admission to the PICU (maximum 7 days after admission). For 14 study subjects, we analyzed twice-weekly samples from admission until discharge from PICU (or maximum of 28 days), with a mean length of stay of 14 days. Relevant clinical variables were recorded at the time of sample collections (see Additional files 1 and 2).

### Sample collection

All samples were collected from PICU and non-PICU study subjects by their nurses at the Children’s Hospital of Pittsburgh (CHP) of the University of Pittsburgh Medical Center under supervision by a single research coordinator (M. S.) to ensure uniformity of sample collection. At all time points, we swabbed the skin (one single pooled sample incorporating the anterior chest wall and both anterior thighs) and tongue dorsum. We also collected fecal samples; when subjects did not provide fecal samples, we obtained a rectal swab. For each body site, the BBL CultureSwab EZ (Becton Dickinson, Franklin Lakes, NJ) was used for sample collection. As described, the composite skin samples were collected by swabbing the anterior chest wall and both anterior thighs. The swabs for skin samples were moistened prior to sample collection with a solution of 0.1% Tween 20 (Sigma Aldrich, St. Louis, MO) and 0.15 M NaCl (Fisher Scientific, Pittsburgh, PA) as described in [44]. Swabs were not moistened for rectal swab and tongue swab collections.

### DNA extraction

All microbial DNA was extracted from clinical samples using the PowerSoil DNA Isolation kit (MO BIO Laboratories, Inc., Carlsbad, CA). Fecal samples were added directly into bead tubes and incubated at 65 °C for 10 min followed by 95 °C for 10 min. After addition of 60 μL of Solution C1, the bead tubes were then shaken horizontally on a lab mixer for 10 min at maximum speed using a MO BIO vortex adaptor. All remaining steps followed the manufacturer’s protocol. For swab samples of the tongue, skin, and rectum, the swab head was cut off directly into bead tubes containing 60 μL of Solution C1 and then incubated at 65 °C for 10 min. Tubes were then shaken horizontally on a lab mixer for 2 min at maximum speed using a MO BIO vortex adaptor. All remaining steps followed the manufacturer’s protocol.

### 16S rRNA gene amplification and sequencing

Bacterial 16S rRNA gene sequences were amplified and sequenced with Roche 454 pyrosequencing. All sequencing runs included appropriate extraction controls and sequencing (not template added) controls (Additional file 8). Amplicons were produced utilizing 338F and 906R primers adapted for the GS FLX Titanium pyrosequencing platform (Roche) to target the V2-V4 region of the 16S rRNA gene. The primers incorporated either the A linker, key, and a 10-nucleotide barcode (forward primer) or the B linker and key (reverse primer), followed by a sequence targeting a conserved region of the bacterial 16S rRNA gene. All individual PCR amplicons were purified, quantified, and pooled in equimolar ratios, and the library pool was gel purified prior to submission for sequencing as described in [45] with the exception that 30 PCR cycles were done. Sequencing was performed on the Roche/454 GS FLX+ System.

### Analysis of 16S rRNA gene sequences

We analyzed our data in combination with samples from the HMP. As the amplicons (V5-V3) generated for the HMP project were sequenced in the opposite direction of ours, we chose a closed OTU picking strategy to generate OTUs using the QIIME pick_closed_reference_otus.py script with the Greengenes database as a reference, resulting in 13,107 predicted OTUs. Taxonomic assignment of the OTUs was done using QIIME 1.8 [46] using green gene database 13.8 and Uclust as a classification method. Any sample with less than 200 reads after OTU picking was not used. OTUs with unassigned taxonomy, or OTUs classified as Streptophytes (potential plant contamination), were also filtered from the OTU table.

### Alpha and beta diversity

Alpha and beta diversity were calculated using QIIME with default parameters and reads rarefied to 500 sequencing reads. Significant differences in alpha diversity were assessed with a non-parametric test using the compare_alpha_diversity.py script in QIIME. Variations in beta diversity were assessed with the PERMANOVA method and algorithms in QIIME. Distance comparisons between adjacent time points, and between samples collected on the same date were done using a custom perl script to parse the distance matrix, and a density plot was generated using the ggplot library in R.

### Identification of differentially abundant taxa within PICU samples

LefsE [31] was used to predict biomarker taxa for each site, comparing across PICU, pediatric, and adult (HMP) samples. Taxa with *p* values below 0.05 (with correction for multiple comparisons) and those with a mean abundance of 5% in either the PICU, pediatric, or adult (HMP) sample groups were retained.

To identify taxa depleted in the PICU dataset, two separate pairwise comparisons were done for each site: between PICU and HMP samples and between PICU and pediatric samples. Significant taxa from these were then compared in R, and the intersection of the comparison (taxa enriched in both the pediatric and HMP vs. PICU) was treated as depleted in the PICU.

## Additional files

Additional file 1: Summary of study population. (XLSX 14 kb)
Additional file 2: Summary of all samples collected from PICU and non-PICU subjects in this study with relevant clinical information. (XLSX 62 kb)
Additional file 3: Principal coordinates analysis plots of weighted UniFrac indices for microbial communities of PICU patients, healthy adults, and healthy children (A, GI; B, skin; C, oral). (D) Principal coordinates analysis plots of microbial communities from all body sites of PICU patients, healthy adults, and healthy children. (E) Principal coordinates analysis plots of microbial communities from all body sites of PICU patients, healthy adults, and healthy children. Here, all PICU samples are highlighted in red regardless of body site. Note the high proportion of centrally located PICU samples that do not fall neatly into the GI, skin, or oral clusters. (PDF 3067 kb)
Additional file 4: Relative abundance of taxonomic groups present at each body site. Taxa included in this display are those that were identified at a relative abundance of greater than 5% at any body site for any group (i.e., either reference groups or PICU). Taxa that were identified by LefSE as biomarkers for the PICU (red), adult (black), or pediatric (blue) groups are marked in the grid below. (PDF 415 kb)
Additional file 5: Relative abundance of taxonomic groups depleted in PICU samples. Taxa included in this display are those that were identified at a relative abundance of greater than 5% at any body site for any group (i.e., either reference groups or PICU). Depleted taxa are defined as those enriched in both the pediatric and the HMP reference groups relative to PICU. (PDF 183 kb)
Additional file 6: Temporal changes in the Shannon diversity index (alpha diversity) of PICU patients. All patients and samples (A, GI; B, skin; C, oral) were included in the primary analyses of alpha diversity, but for simplicity, only patients with three or more samples per body site are shown here. (PDF 5 kb)
Additional file 7: Density plots of binary Jaccard distances of samples from the same subject on adjacent sampling dates (yellow) and samples collected on the same study day from different individuals (blue). At all sites, the median differences (indicated by dotted lines) between adjacent dates from the same sample were far smaller than those taken from different subjects on the same study day. However, the overlap of the curves indicates that some sample sets from the same subject differed sharply despite collection on adjacent study dates. (PDF 347 kb)
Additional file 8: Relative abundance of taxonomic groups identified in extraction controls (labeled as EC) and no template added negative (labeled as NTC) controls. (PDF 56 kb)
