# Supplementary figures and images for: Disruption of the microbiota across multiple body sites in critically ill children
Source: Microbiome. 2016 Dec 29;4:66. doi: 10.1186/s40168-016-0211-0 (PMC5200963; doi:10.1186/s40168-016-0211-0)

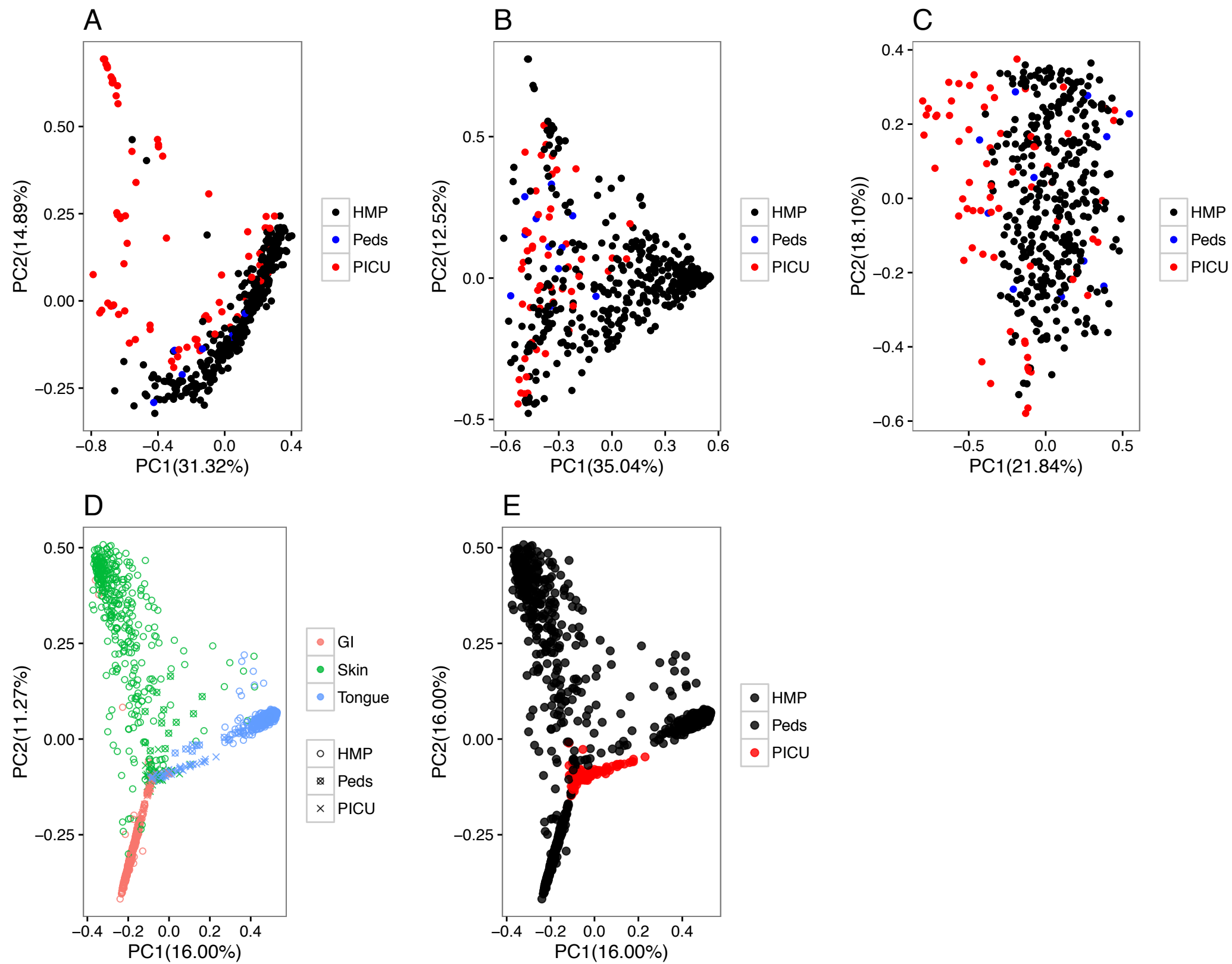

Supplement: Additional file 3: — Principal coordinates analysis plots of weighted UniFrac indices for microbial communities of PICU patients, healthy adults, and healthy children (A, GI; B, skin; C, oral). (D) Principal coordinates analysis plots of microbial communities from all body sites of PICU patients, healthy adults, and healthy children. (E) Principal coordinates analysis plots of microbial communities from all body sites of PICU patients, healthy adults, and healthy children. Here, all PICU samples are highlighted in red regardless of body site. Note the high proportion of centrally located PICU samples that do not fall neatly into the GI, skin, or oral clusters. (PDF 3067 kb) [file 40168_2016_211_MOESM3_ESM.pdf]

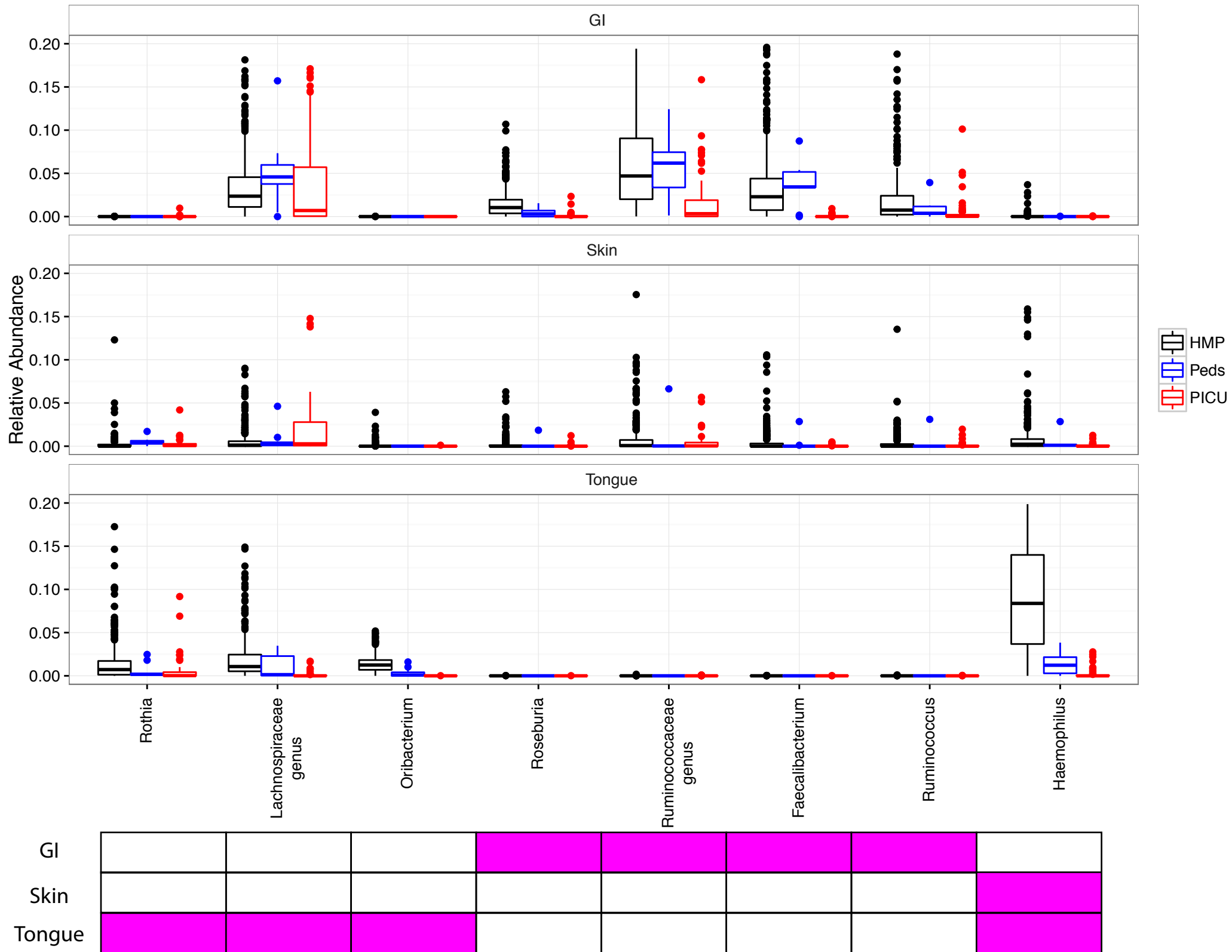

Supplement: Additional file 5: — Relative abundance of taxonomic groups depleted in PICU samples. Taxa included in this display are those that were identified at a relative abundance of greater than 5% at any body site for any group (i.e., either reference groups or PICU). Depleted taxa are defined as those enriched in both the pediatric and the HMP reference groups relative to PICU. (PDF 183 kb) [file 40168_2016_211_MOESM5_ESM.pdf]

**A**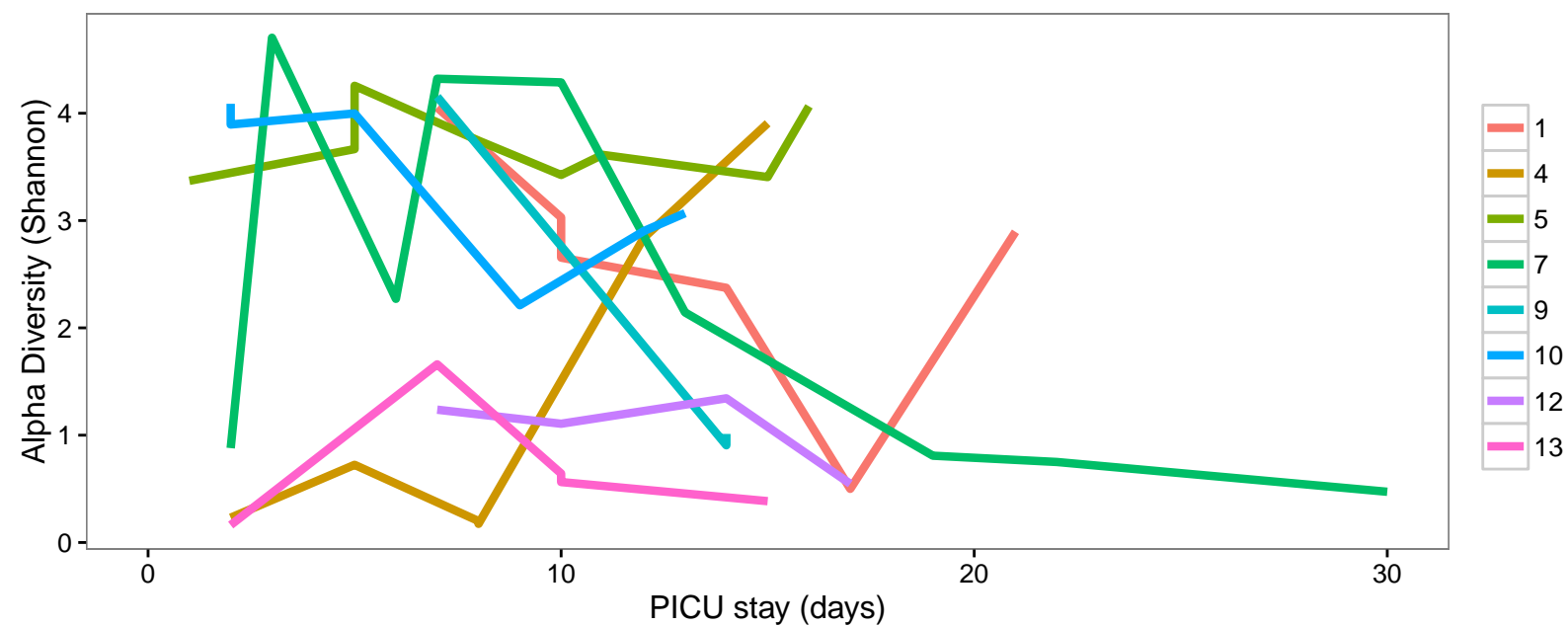**B**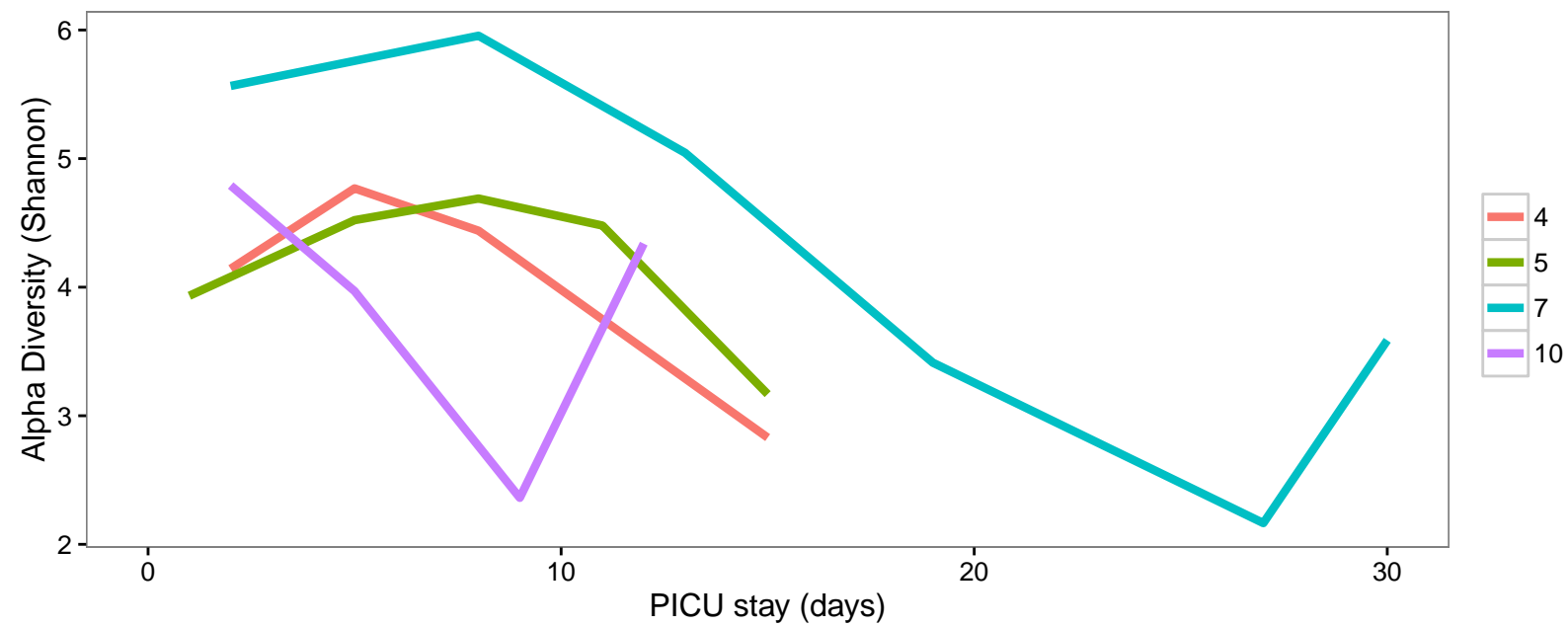**C**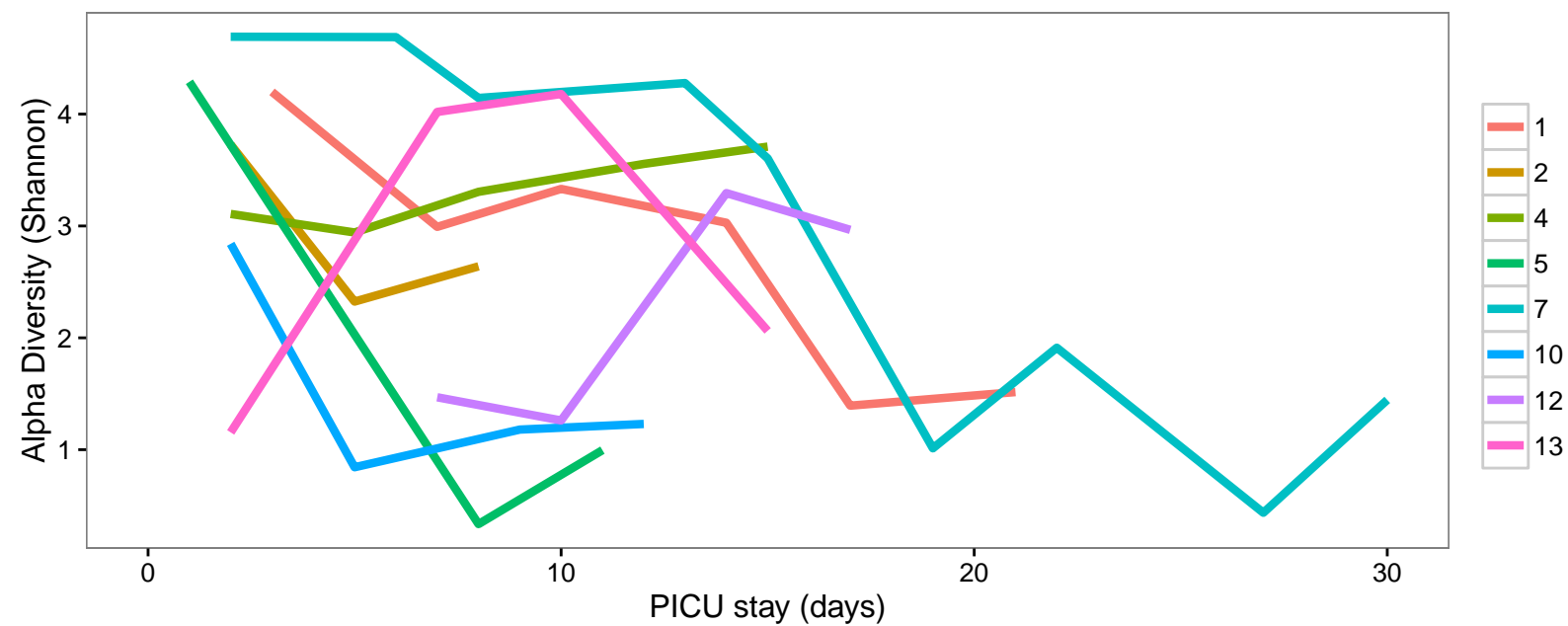

Supplement: Additional file 6: — Temporal changes in the Shannon diversity index (alpha diversity) of PICU patients. All patients and samples (A, GI; B, skin; C, oral) were included in the primary analyses of alpha diversity, but for simplicity, only patients with three or more samples per body site are shown here. (PDF 5 kb) [file 40168_2016_211_MOESM6_ESM.pdf]

GI Samples

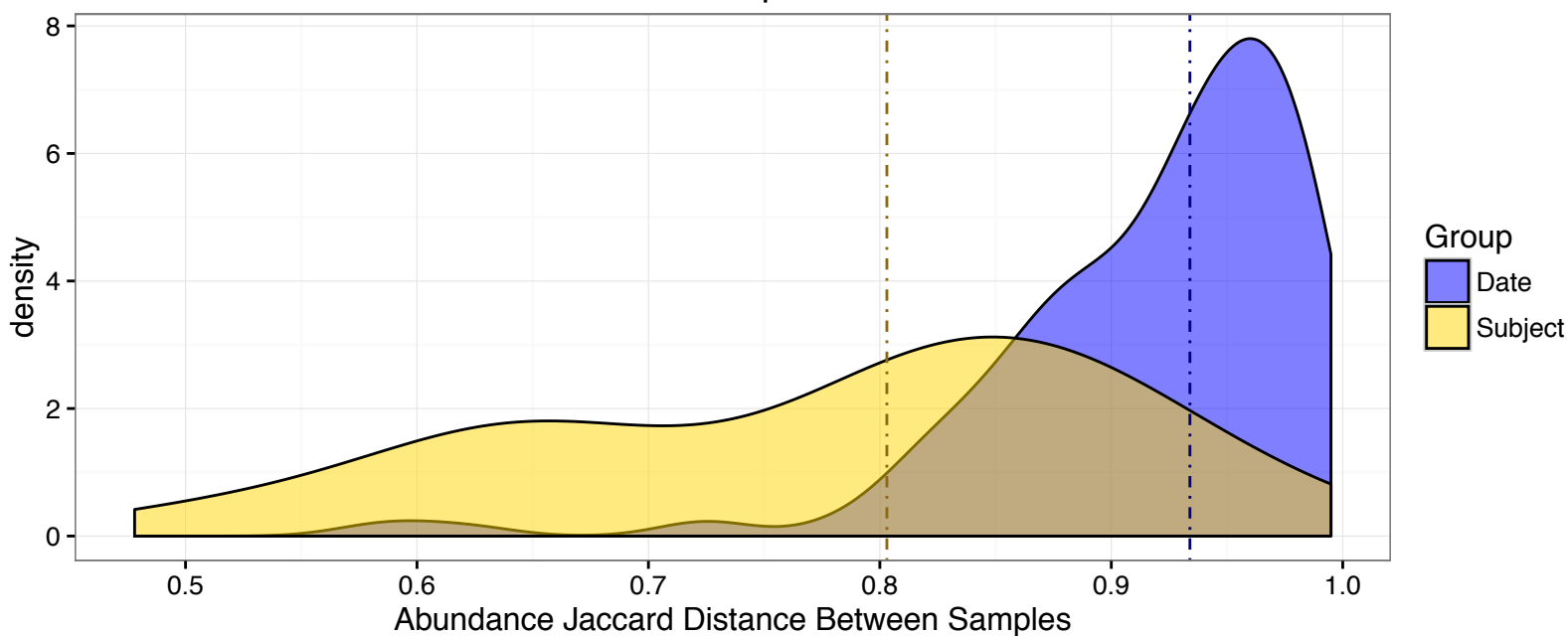

Oral Samples

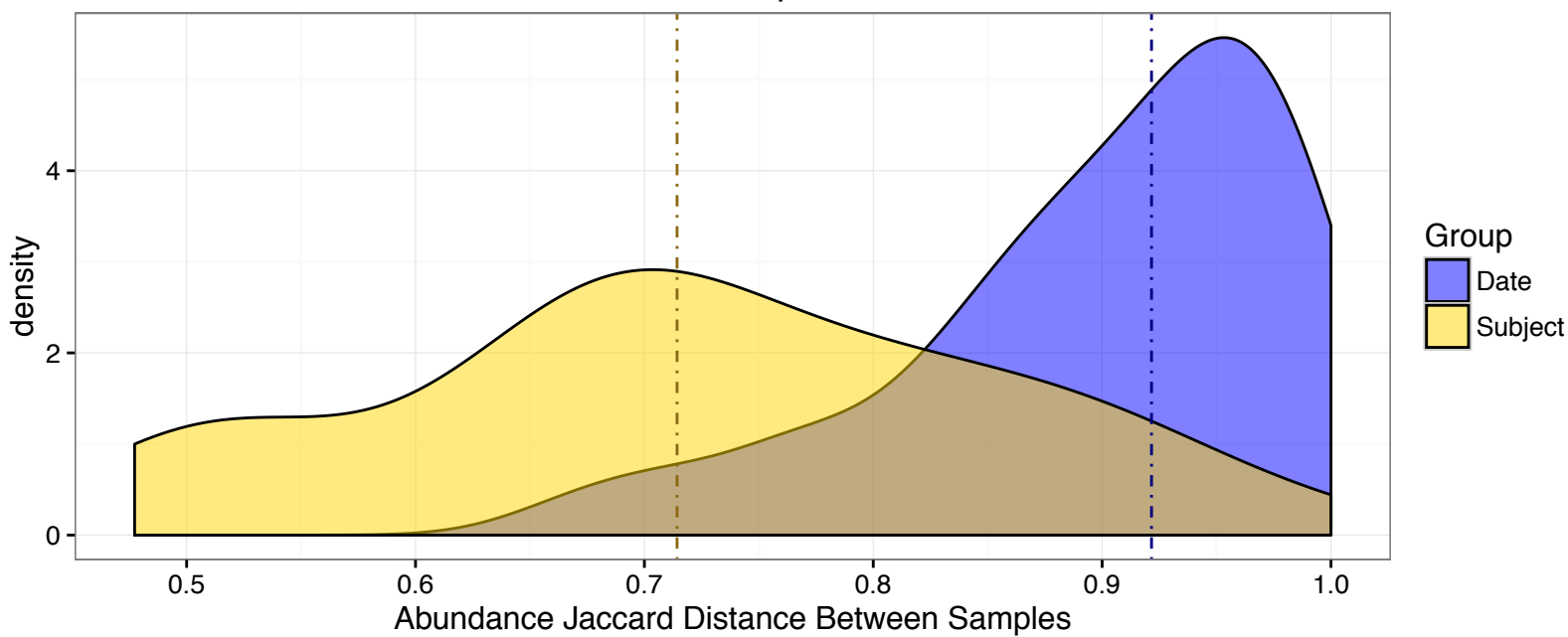

Skin Samples

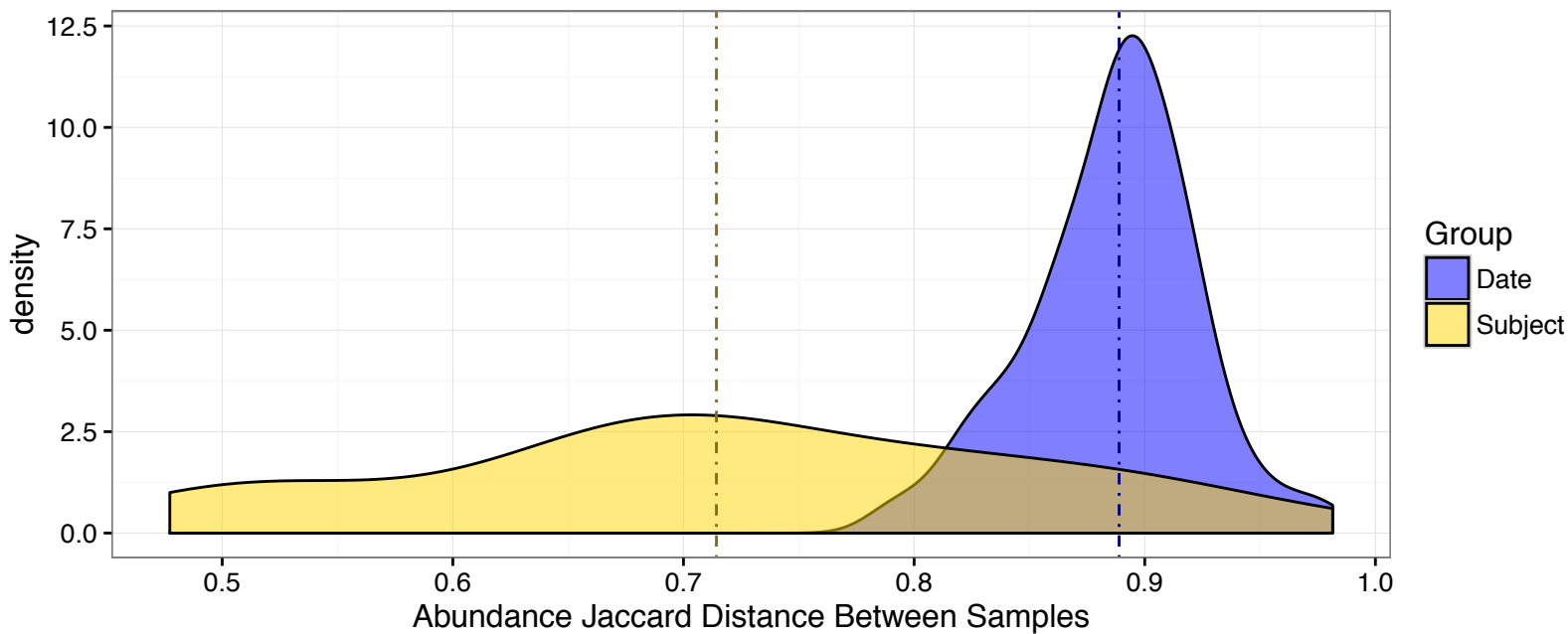

Supplement: Additional file 7: — Density plots of binary Jaccard distances of samples from the same subject on adjacent sampling dates (yellow) and samples collected on the same study day from different individuals (blue). At all sites, the median differences (indicated by dotted lines) between adjacent dates from the same sample were far smaller than those taken from different subjects on the same study day. However, the overlap of the curves indicates that some sample sets from the same subject differed sharply despite collection on adjacent study dates. (PDF 347 kb) [file 40168_2016_211_MOESM7_ESM.pdf]

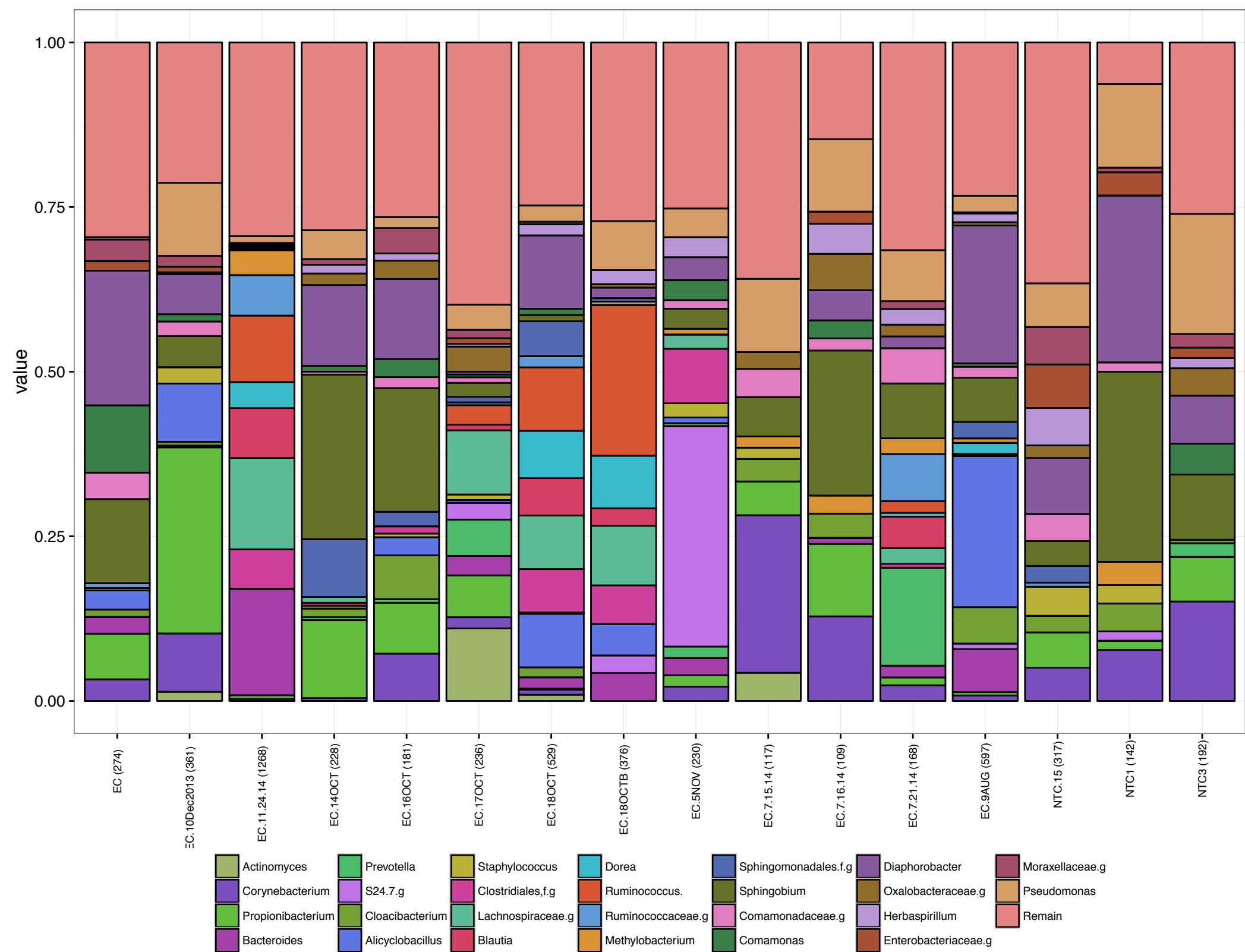

Supplement: Additional file 8: — Relative abundance of taxonomic groups identified in extraction controls (labeled as EC) and no template added negative (labeled as NTC) controls. (PDF 56 kb) [file 40168_2016_211_MOESM8_ESM.pdf]
