# Supplementary material for: Disruption of the microbiota across multiple body sites in critically ill children
Source: Microbiome. 2016 Dec 29;4:66. doi: 10.1186/s40168-016-0211-0 (PMC5200963; doi:10.1186/s40168-016-0211-0)
Supplement: Additional file 4: — Relative abundance of taxonomic groups present at each body site. Taxa included in this display are those that were identified at a relative abundance of greater than 5% at any body site for any group (i.e., either reference groups or PICU). Taxa that were identified by LefSE as biomarkers for the PICU (red), adult (black), or pediatric (blue) groups are marked in the grid below. (PDF 415 kb) [file 40168_2016_211_MOESM4_ESM.pdf]

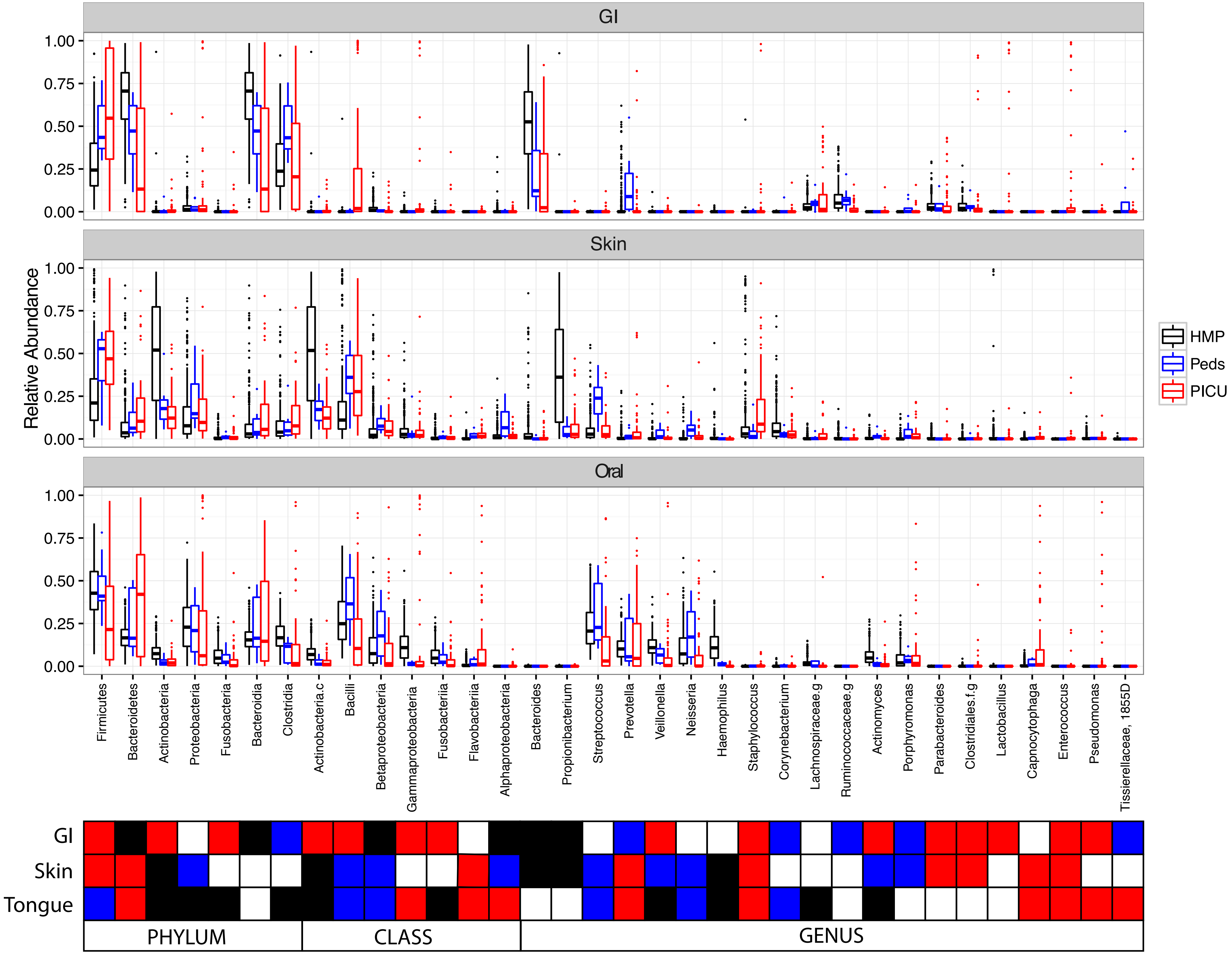
GI
Skin
Oral
Relative Abundance
0.00
0.25
0.50
0.75
1.00
HMP
Peds
PICU
Firmicutes
Bacteroidetes
Actinobacteria
Proteobacteria
Fusobacteria
Bacteroidia
Clostridia
Actinobacteria.c
Bacilli
Betaproteobacteria
Gammaproteobacteria
Fusobacteriia
Flavobacteriia
Alphaproteobacteria
Bacteroides
Propionibacterium
Streptococcus
Prevotella
Veillonella
Neisseria
Haemophilus
Staphylococcus
Corynebacterium
Lachnospiraceae.g
Ruminococcaceae.g
Actinomyces
Porphyromonas
Parabacteroides
Clostridiales.f.g
Lactobacillus
Capnocytophaga
Enterococcus
Pseudomonas
Tissierellaceae, 1855D
GI
Skin
Tongue
PHYLUM
CLASS
GENUS
